# Supplementary material for: Identification of photoperiod-induced specific miRNAs in the adrenal glands of Sunite sheep (Ovis aries)
Source: Front Vet Sci. 2022 Jul 22;9:888207. doi: 10.3389/fvets.2022.888207 (PMC9354845; doi:10.3389/fvets.2022.888207)
Supplement: Supplementary file 1 [file Data_Sheet_1.ZIP › Supplementary file/miRDeep2 file.zip!/miRDeep2 file/Know_Structure/SP42A3/oar-mir-323a.pdf]

miRBase precursor : oar-mir-323a
Total read count : 941
oar-miR-323a-5p read count : 5
oar-miR-323a-3p read count : 935
remaining reads : 1

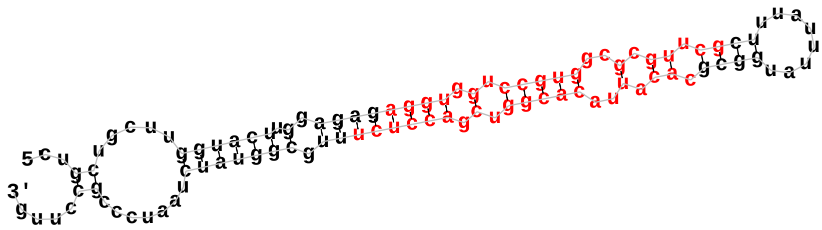

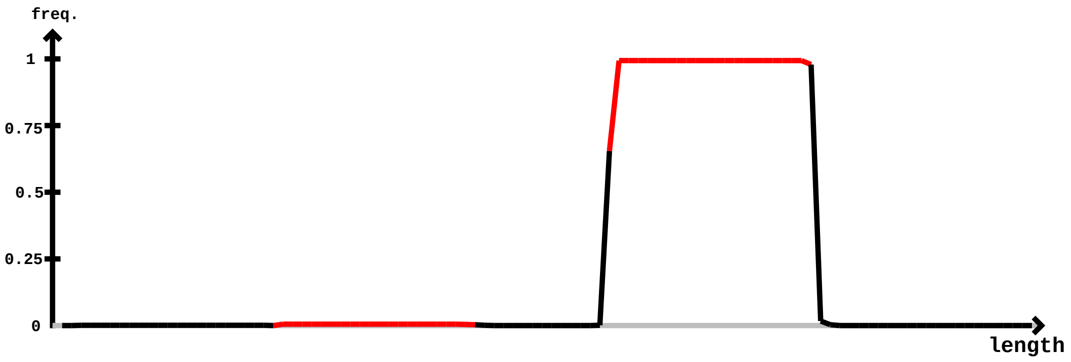


```
                           oar-miR-323a-5p
                                                  oar-miR-323a-3p
5'- cugcugcuugguacuuggagagaggugguccguggcgcguucgcuuuauuuauggcgcacauuacacggucgaccucuuugcgguaucuaaucccgccuug   -3'    exp
    .........(((((.(.((((((((.(.(((((..(.((.((((........)))).)).)..))))).).)))))))).)))))))............   reads   mm   sample
    .ugcugcuugguacuuggaga.................................................................................   1       0    seq
    ......................agguggucc guggcgcguu.............................................................   1       0    seq
    ......................aggugguccguggcgcguuc............................................................   1       0    seq
    ......................aggugguccguggcgcguucg...........................................................   2       0    seq
    ......................aggugguccguggcgcguucgc..........................................................   1       0    seq
    ........................................................cgcacauuacacggucgaccucu.......................   1       0    seq
    .........................................................gcacauuacacggucgaccuc........................   12      0    seq
    .........................................................gcacauuacacggucgaccucu.......................   598     0    seq
    .........................................................gcacauuacacggucgaccucuu......................   4       0    seq
    .........................................................gcacauuacacggucgaccucuuu.....................   1       0    seq
    ..........................................................cacauuacacggucgaccuc........................   2       0    seq
    ..........................................................cacauuacacggucgaccucu.......................   306     0    seq
    ..........................................................cacauuacacggucgaccucuu......................   9       0    seq
    ..........................................................cacauuacacggucgaccucuuu.....................   2       0    seq
```
